# Liver Resection Promotes (Regulates) Proinflammatory Cytokines in Patients with Hepatocellular Carcinoma

**DOI:** 10.1155/2021/5593655

**Published:** 2021-04-23

**Authors:** Farshid Fathi, Behnam Sanei, Mazdak Ganjalikhani Hakemi, Reza F. Saidi, Abbas Rezaei

**Affiliations:** ^1^Department of Immunology, School of Medicine, Isfahan University of Medical Sciences, Isfahan, Iran; ^2^Department of Hepatobiliary & Pancreatic Surgery and Liver Transplantation, Al-Zahra Hospital, Isfahan University of Medical Sciences, Isfahan, Iran; ^3^Division of Transplant Services, Department of Surgery, SUNY Upstate Medical University Syracuse, Syracuse, NY 13210, USA

## Abstract

**Background:**

Several animal studies have shown the roles of cytokines in regulating liver regeneration following liver resection (LR), which is a type of surgery designed to remove cancerous tumors from the liver. This study investigated how the expressions and serum levels of some pro- and anti-inflammatory cytokines in patients with hepatocellular carcinoma (HCC) were changed during LR.

**Methods:**

Liver tissues from 15 patients with HCC were collected and the levels of interleukin-6 (IL-6), tumor necrosis factor-alpha (TNF-*α*), IL-1*α*, IL-1 *β*, IL-10, and transforming growth factor-beta1 (TGF-*β*1) were assessed using real-time PCR assay at different times before and after LR. The serum values of TNF-*α* and IL-6 were also measured by ELISA.

**Results:**

After 60 and 90 minutes of LR, IL-6 gene expression was significantly increased (*P* < 0.001 − 0.05). The same trend was also observed in TNF-*α* expression after 90 minutes of LR (*P* < 0.01). No significant changes were observed in the expressions of IL-1*α*, IL-1*β*, IL-10, and TGF-*β*1 before and after LR. In addition, LR had significant effects on TNF-*α* and IL-6 serum levels (*P* < 0.05 − 0.0001).

**Conclusion:**

Our data provided further evidence to reveal that IL-6 and TNF-*α* cytokines are critical to improve liver regeneration.

## 1. Introduction

Hepatocellular carcinoma (HCC) is one of the most common malignant tumors worldwide which predominantly occurs in subjects with chronic liver diseases [1]. HCC is responsible for approximately 1000000 deaths annually, making it the third leading cause of cancer death [2]. Recent evidence from different countries has shown that the incidence of HCC is rising [3–5]. Its causes have not been identified yet, but some factors are proposed for increasing the risk for the susceptibility, including hepatitis B and C infections, liver cirrhosis, alcohol consumption, obesity and diabetes, iron storage disease, aflatoxin toxicity, fatty liver disease, and drug-induced liver injury [1]. The initiation and progression of this disease are associated with genetic, environmental, and immunological factors. Some therapies have been proposed to treat HCC, including liver resection (LR), liver transplantation, stem cell transplantation, and artificial liver support systems [6]. In patients who were diagnosed at early stages (Barcelona Clinic Liver Cancer Stage 0 or A—BCLC 0/A), LR and local ablation are potentially effective and increase life expectancy remarkably [2].

In normal condition, hepatocytes and cholangiocytes stay in the G0 phase of the cell cycle and indicate minimal turnover, but in response to various stimulations such as toxic injury, infections, and LR, they proliferate to compensate for the lost cells, a process called liver regeneration [7, 8]. Although the precise mechanism of liver regeneration is not well understood, extensive data from the literature have demonstrated that the immune system plays an important role in the initiation and termination of liver regeneration through pro- and anti-inflammatory mediators such as cytokines, complement, and chemokines [9, 10]. Several studies have shown that cytokines stimulate hepatocytes to start the cell cycle [11, 12]. Others have indicated that cytokines contribute to recruit immune cells such as T cells, B cells, macrophages, NKT, and NK cells into liver tissue to improve liver regeneration [13]. Several cytokines and chemokines such as IL-1, IL-6, TNF-*α*, IL-10, TGF-*β*, CCL2, and CCL5 have been reported for their contributions in liver regeneration [14–16].

Some studies have emphasized the roles of IL-6 and soluble IL-6 receptor in liver regeneration [17]. Some animal studies have revealed that IL-6-deficient mice had liver failure and defective hepatocyte regeneration in which a single preoperative dose of IL-6 prevented liver damages through returning gene expression and hepatocyte proliferation to near normal [16]. Moreover, it is demonstrated that IL-6 and TNF-*α* had pivotal roles in early graft regeneration in patients who underwent living donor liver transplantation (LDLT) [18]. These findings suggest a possible therapeutic potential for IL-6 and TNF-*α* to recover liver functions in clinical situations associated with liver regeneration such as acute hepatic failure or resection of chronically damaged liver tissue.

Some cells have been proposed as the cell sources of the most important cytokines for liver regeneration, including hepatocytes, hepatic stellate cells, infiltrated immune cells, and cancer-associated fibroblasts [10]. Numerous reports have indicated the role of pro- and anti-inflammatory cytokines in liver regeneration in animal studies [19]. To our knowledge, there is no report showing the expression levels of these cytokines in patients with HCC after LR. This study was therefore focused on investigating the gene expressions of IL-1*α*, IL-1*β*, IL-6, and TNF-*α* as pro-inflammatory and TGF-*β*1 and IL-10 as anti-inflammatory cytokines in patients with HCC before and after LR.

## 2. Material and Methods

### 2.1. Study Population

The study population consisted of 15 individuals referred to surgery centers of Al-Zahra and Sina hospitals in Isfahan and Tehran, respectively. This work was conducted from April 2019 to April 2020. The HCC stage was determined by a specialist according to the eighth edition of the Cancer Staging Manual by the American Joint Committee on Cancer (AJCC) [20]. All these patients have well-compensated cirrhosis with normal liver function tests. The sampling was carried out at least one month after the last radiotherapy, chemotherapy, and other therapeutic approaches. None of the patients was on preoperative radiotherapy, chemotherapy, and other medical interventions, which could affect the immune system and cytokine productions at the time of the sampling. Experimental protocols were approved by the Ethics Committee of Tehran University of Medical Sciences (ethic code: IR.TUMS.VCR.REC1396.4790) and performed according to the Declaration of Helsinki. All participants were informed before entering the study and informed consent was obtained from the subjects.

### 2.2. Sample Collection

LR was performed by a surgical team, and approximately 70% of the total liver was removed. Liver tissues were collected from patients several times following surgical resection. Briefly, in the first place, the sample was obtained after starting surgery opened the abdominal cavity and before LR. Malignant tissues were removed and remained liver tissue was subjected to sampling (30, 60, and 90 minutes after LR). The samples were stored at −196°C for the next experiments.

### 2.3. RNA Extraction and Complementary Deoxyribonucleic Acid (cDNA) Synthesis

The total RNAs were isolated from the frozen liver tissues using an RNA isolation kit based on the manufacturer's instructions (Yekta Tajhiz, Iran). The quantity and purity of the extracted RNAs were determined by NanoDrop (Thermo Fisher Scientific, USA) and run in 1% agarose gel. The cDNA synthesis was performed using a reverse transcription kit (Biofact, South Korea) according to the manufacturer's instructions. Briefly, the first strand of cDNA was synthesized in the total volume of 10 *μ*l, containing 1 *μ*l (1 ng) of mRNA, 1 *μ*l of RT enzyme, 4 *μ*l of 10X buffer, 2 *μ*l of random hexamer and poly-A (dT) primers, and 2 *μ*l of the nuclease-free water. Then, the reaction mixture was incubated at 42°C for 1 hour. The cDNA was stored at −80°C for use in real-time polymerase chain reaction (PCR).

### 2.4. Real-Time PCR

To assess the mRNA levels of IL-1*α*, IL-1*β*, IL-6, IL-10, TNF-*α*, and TGF-*β*, real-time PCR was carried out using an ABI7700 machine (Applied Biosystems, Foster City, USA). Each reaction was initiated at 95°C for 10 minutes followed by 40 cycles of 95°C for 30 seconds and 60°C for 40 seconds. All analyses were done in triplicate. Real-time PCR was carried out in a reaction mixture (10 *μ*L) consisted of 4 *μ*L of 2X Real-Time PCR master mix (SYBR® Premix Ex Taq™ II; Bio Fact, Korea), 2 *μ*L of forward and reverse primers (10 pM), 1 *μ*L of cDNA template, 2.7 *μ*L of DNase-RNase free water, and 0.3 *μ*L of ROX. The temperature and cycling parameters mentioned above were used to determine the expression levels of IL-1*α*, IL-1*β*, IL-6, IL-10, TNF-*α*, and TGF-*β*1. The melting curves were automatically generated in the 60°C to 95°C temperature range. The primer sequences and other information of real-time PCR are shown in Table 1. The expression levels of the genes were normalized to the expression levels of *β*-actin and glyceraldehyde-3-phosphate dehydrogenase (GAPDH) genes as the endogenous controls. The expression levels of the genes were calculated according to the 2^−∆∆CT^ method [21]. The primers were designed by AlleleID 7.5 software (Premier Biosoft Intl, Palo Alto, CA, USA) and purchased from Takapou Zist (Iran).

### 2.5. Cytokine Assay

To evaluate the effect of LR on the serum levels of cytokines in HCC patients, peripheral blood samples were obtained from patients before and after 30, 60, 90, and 180 minutes of LR. The serum levels of TNF-*α* and IL-6 were measured using an enzyme-linked immunosorbent assay (ELISA) kit according to the manufacturer's protocol (Karmania Pars Gene, Iran). All assays were performed in duplicate.

### 2.6. Statistical Analysis

Data analysis was performed by GraphPad Prism 6 (GraphPad Software, USA). The results are shown as the standard error of the mean (SEM). The normal distributions of the data were determined by Kolmogorov–Smirnov test. One-way ANOVA and paired *t*-tests were used to compare the groups with normal distribution. Data with nonparametric distribution were analyzed using Mann–Whitney and Kruskal–Wallis tests. The significance level was considered as *P* < 0.05.

## 3. Results

### 3.1. Subject Descriptions

A total of 15 individuals with HCC (8 males and 7 females, mean age: 60 ± 2.59, mean ± standard deviation, range: 56 to 66 years) were enrolled in this study (Table 2). All patients with HCC had a primary tumor and most of them were in stage IIIA (46.66%, Table 2). According to Barcelona Clinic Liver Cancer (BCLC) system, all our patients were in 0 or A stage with a single tumor or up to 3 nodules smaller than 3 cm or Child–Pugh–Turcotte (CPT A). Table 2 depicts the clinicopathological characteristics of HCC subjects.

### 3.2. The Impacts of LR on the Expression Levels and Serum Levels of Proinflammatory Cytokines in HCC Patients

**Figure 1:**
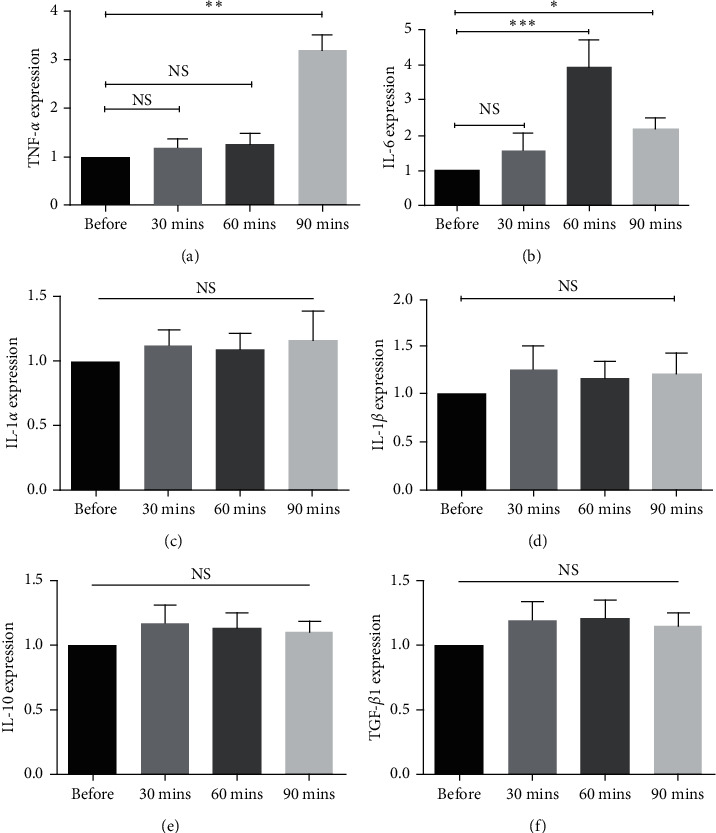
The effect of liver regeneration on the expression levels of proinflammatory and anti-inflammatory cytokines in HCC patients. The expressions of (a) IL-6, (b) TNF-*α*, (c) IL-1*α*, (d) IL-1*β*, (e) IL-10, and (f) TGF-*β*1 were determined by real-time PCR assay before and after LR. All data are shown as mean ± SEM. The depicted results are representative of 15 independent experiments. NS indicates that the differences in the expression levels are not statistically significant. Asterisks indicate that the differences in the expression levels are statistically significant. ^*∗*^*P* < 0.05, ^*∗∗*^*P* < 0.01, and ^*∗∗∗*^*P* < 0.001.

To determine the effect of LR on the expression levels of TNF-*α*, IL-1*α*, IL-1*β*, and IL-6, the levels of these cytokines were assessed several times using the real-time PCR method. The results showed that the expressions of IL-6 and TNF-*α* were increased after LR. After 60 and 90 minutes of LR, IL-6 gene expression was significantly increased (*P* < 0.001 − 0.05, Figure 1(a)). The means of expression changes were 3.9- and 2.1-fold after 60 and 90 minutes of LR, respectively (Figure 1(a)). The same trend was also observed in TNF-*α* expression after 90 minutes of LR (*P* < 0.01, Figure 1(b)). The mean of expression change was 3.2-fold after 90 minutes. No significant changes in the expressions of IL-1*α* and IL-1*β* were observed before and after LR (Figures 1(c) and 1(d)).

### 3.3. The Expression Levels of Anti-Inflammatory Cytokines in HCC Patients before and after LR

**Figure 2:**
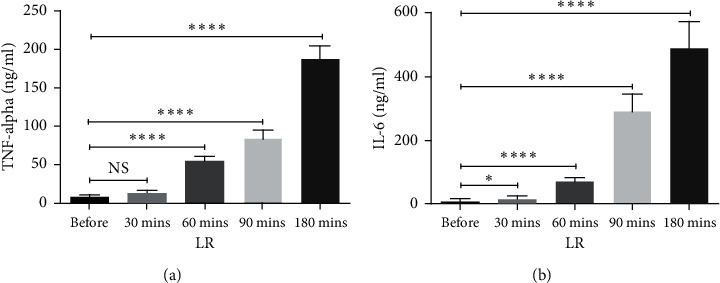
The effect of liver regeneration on the serum concentrations of proinflammatory cytokines in HCC patients. The levels of TNF-*α* and IL-6 in patients with HCC were measured before and after liver regeneration using ELISA ((a) and (b)). The depicted results are representative of 15 independent experiments for HCC subjects. All data are shown as mean ± SEM. ^*∗*^*P* < 0.05 and ^*∗∗∗*^*P* < 0.001.

The results of the real-time PCR method indicated that LR did not affect IL-10 and TGF-*β*1 expression levels. There were no significant differences in the expression levels of these cytokines before and after LR (Figures 2(e) and 2(f)).

### 3.4. The Impact of LR on the Serum Levels of Proinflammatory Cytokines

To further confirm the possible effect of LR on expression levels of cytokines, the serum levels of cytokines, whose expressions were affected by LR, were studied before and after 30, 60, 90, and 180 minutes of LR. Similar trends were observed in the serum values of TNF-*α* and IL-6 (Figures 2(a) and 2(b)). LR had a potent impact on elevating TNF-*α* and IL-6 levels (*P* < 0.05 − 0.0001, Figures 2(a) and 2(b)).

## 4. Discussion

Liver regeneration is a process to return to the normal size of the liver after injury and surgery [22]. Under normal condition, cell turnover rarely occurs in the liver, but two-thirds LR, as the most common method to remove malignant liver tumors, induces the proliferation of mature hepatocytes [23–25]. Although there are several animal studies pointing to the roles of cytokines in liver regeneration after LR [26], this study, for the first time, investigated how LR influences the gene expressions of IL-1*α*, IL-1*β*, IL-6, TNF-*α*, IL-10, and TGF-*β* in HCC patients.

Our results indicated that the expressions and serum levels of IL-6 and TNF-*α* after LR were significantly increased in comparison with their levels before LR. Transcript levels of IL-6 and TNF-*α* were significantly increased after 60 and 90 minutes of LR, respectively. In agreement with these findings, previous studies have revealed that the serum levels of hepatocyte growth factor (HGF) and IL-6 increased at similar rates during the first 24 hours after LR in patients with focal nodular hyperplasia and hepatic hemangioma [27]. Several studies have declared that inflammatory responses induced upon LR were immediately needed for liver regeneration [28]. In line with this notion, animal studies have shown that approximately 70% of IL-6 gene knocking out mice failed to have liver regeneration [29]. It is reported that Kupffer cells as a tissue-resident macrophage are the main source of cytokines associated with liver regeneration, including IL-6, TNF-*α*, IL-10, and TGF-*β*1 [30–32]. Therefore, these cells are critical for hepatocyte regeneration after LR. Animal studies have revealed that Kupffer cell depletion affects hepatic IL-6, IL-10, TNF-*α*, and TGF-*β*1 mRNA syntheses and thereby delays liver regeneration after LR [26]. Yasuhiro et al. showed that knock out of TNFR-1 gene led to defect in liver regeneration after CCL4-induced acute liver failure [33]. These findings are consistent with our results pointing to the necessity of IL-6 and TNF-*α* for liver regeneration. However, they are in contrast with other studies showing the depletions of proinflammatory genes did not affect liver regeneration. An animal study conducted by Fujita et al. declared that TNF gene depletion had no effect on liver regeneration [34]. However, it negatively affected liver function and neutrophil activation [34]. Other results of the current study revealed there are no significant differences in gene expressions of IL-1*α*, IL-1*β*, IL-10, and TGF-*β*1 before and after LR. In contrast to our data, some animal studies have demonstrated the role of IL-1 in liver regeneration after LR. Ma et al. indicated that downregulation of IL-1*β* gene by siRNA could improve liver regeneration in rates after CCL4-induced acute liver failure [35]. Another study on rats has shown that the reduced serum level of IL-1*β* promoted liver regeneration after liver failure [36]. In addition, others have revealed that IL-1 receptor antagonist (IL-1R*α*) significantly reduced inflammation after LR and enhanced liver regeneration [37]. Moreover, LR is the mainstay of curative treatment and health-giving treatment for HCC, and the long-term survival of patients still remains undesirable since recurrence happens in about 54% of patients who underwent resection [38, 39]. Recurrence after LR is highly associated with aggressive tumor characteristics, size of the tumor, presence of cirrhosis, Child–Pugh classification, alcohol consumption, and smoking [40].On the other hand, all HCC patients are not suitable for LR [41]. Ascites and uncorrectable coagulopathy are two major factors that reduce the number of candidates for surgery; furthermore, radiofrequency ablation has been suggested by the researcher as an alternative therapy for these CPT A and B HCC patients, and 3-year survival of patients reached up to 65% after it [42]. LR and radiofrequency ablation liver transplantation is a curative option for HCC patients, and there are types of adjuvant therapy for CPT A and B HCC patients, for instance, Transarterial chemoembolization (TACE), transarterial radioembolization (TARE), sorafenib, antiviral therapy, and IFN [42, 43]. Although sorafenib is the only approved drug for HCC treatment, a notable number of sorafenib-treated patients experience disease progression. Furthermore, investigators introduced second-line systemic therapy instead of sorafenib [44]. Capecitabine (Xeloda®, Roche) is a replacement for sorafenib, and studies by Granito et al. showed that it is a safe and effective treatment as a second-line therapy in patients unresponsive to sorafenib [45]. On the other hand, Xia et al. showed adjuvant therapy with capecitabine after LR significantly decreases the risk of HCC [46]. Although patient's outcome is critical after LR, in this study we did not follow up the patients to access the survival or recurrence rate and our focus was regeneration.

Also in an attempt to determine the effect of LR on the expression levels of cytokines involved in controlling inflammation, the levels of IL-10 and TGF-*β*1 were investigated. In contrast with other reports showing the levels of anti-inflammatory cytokines were increased following LR, the results of the present study showed that LR had no significant effect on TGF-*β* and IL-10 expressions. Shin et al. in a study on rats reported that IL-10 expression along with other cytokines (TNF, IL-1, and IL-6) significantly increased within three hours after LR [47]. Furthermore, the authors have revealed that inhibition of IL-10 production by Kupffer cell depletion enhanced the expression of TNF-*α* in the regeneration of rat liver [47] and induced a cytokine cascade which promoted hepatocyte proliferation after 70% LR. In line with the expression levels of other anti-inflammatory cytokines after LR, Picardo et al. indicated that the increase in TGF-*β* mRNA level occurred following TNF-*α*, IL-6, and HGF after LR [48]. TGF-*β*1, like IL-10, exerts an inhibitory effect on hepatocyte proliferation and thereby plays an important role in the termination of liver regeneration [9, 25, 48]. It is reported that TGF-*β* signaling in hepatocytes inhibited the proliferative responses after LR [49]. Others have revealed that TGF-*β*1 inhibition with anti-TGF-*β*1 antibody after LR in dimethylnitrosamine-treated cirrhotic and biliary-obstructed rats improved liver regeneration both morphologically and functionally [50, 51]. Therefore, the reduction in the levels of TGF-*α* and TGF-*β* receptors following LR may be a compensatory response to enhance liver regeneration [52]. However, these discrepancies observed between our results and previous studies may be related to the sample sizes, sample types, and sampling times used in different studies.

## 5. Conclusion

The results of the current study, consistent with animal studies, provide further evidence to reveal that the production of proinflammatory cytokines was increased after LR and may have a role in regulating liver regeneration. However, a limitation of the study was the lack of healthy subjects as a control group. Therefore, this limitation should be considered to confirm these findings in future studies.

## Figures and Tables

**Table 1:** Primer sequences and other information used to determine the expressions of pro- and anti-inflammatory cytokines.

Gene	Primer sequence	Amplicon size (bp)	Annealing temperatures (°C)
IL-1*α*	Forward (5′-3′) GTGACTGCCCAAGATGAAGACC Reverse (5′-3′) TCCCAGAAGAAGAGGAGGTTGG	101	61

IL-6	Forward (5′-3′) AGAGTAACATGTGTGAAAGCAGCA Reverse (5′-3′) TGATGATTTTCACCAGGCAAGTCT	129	61

IL-1*β*	Forward (5′-3′) AGGGACAGGATATGGAGCAACA Reverse (5′-3′) CTTTCAACACGCAGGACAGGT	129	61

IL-10	Forward (5′-3′) TCAAGGCGCATGTGAACTCC Reverse (5′-3′) CATTCTTCACCTGCTCCACGG	120	61

TGF-*β*1	Forward (5′-3′) CATGCCAACTTCTGCCTCGG Reverse (5′-3′) TGGTTGTACAGGGCCAGGAC	83	61

TNF-*α*	Forward (5′-3′) GACCTCTCTCTAATCAGCCCTCT Reverse (5′-3′) CTGGTTATCTCTCAGCTCCACG	174	61

**Table 2:** The clinicopathological characteristics of participants.

Age (mean ± SD)	60 ± 2.59
Gender	Female: 7 (46.66) Male: 8 (53.33)
Tumor type	HCC: 15 (100%)
TNM*∗* Primary tumor Regional lymph nodes Distant metastasis	T2: 4 (26.66%); T3: 7 (46.66%); T4: 4(26.66%) 0 (0.0%) 0 (0.0%)
Stage	II: 4 (26.66%) IIIA: 7 (46.66%) IIIB: 4 (26.66%)
Tumor size	<2 : 6 (40%) ≤2–5 ≤: 7 (46.66%) 5 >: 2 (13.34%)
Hepatitis B	2 (13.34%)
Smoking history	6 (40%)
Alcohol consumption	44(26.66%)
WBC	9766 ± 1240
Hb (g/dl)	11.67 ± 1.54
Hct (%)	39.45 ± 3.18
FBS (mg/dl)	96.11 ± 7.9
Creatinine (mg/dl)	0.96 ± 0.16
BUN (mg/dl)	15.56 ± 3.19
Urea (mg/dl)	9.67 ± 2.54
AST (U/L)	36.43 ± 3.12
ALT(U/L)	34.8 ± 4.39
Albumin (g/dl)	3.86 ± 0.81
Total bilirubin (mg/dl)	1.06 ± 0.4
PT (seconds)	13.5 ± 1.67
PTT (seconds)	34.1 ± 3.89

*∗*TNM staging based on AJCC 8th edition. HCC: hepatocellular carcinoma; Hb: hemoglobin; Hct; hematocrit; FBS: fasting blood sugar; BUN: blood urea nitrogen; AST: aspartate aminotransferase; ALT: alanine transaminase; PT: prothrombin time; PTT: partial thromboplastin time.

## Data Availability

The data used to support the findings of this study are available from the corresponding author upon request.
